# Anthrax and Preventive Inoculation in Louisiana1Read before the Thirty-eighth Annual Meeting of the American Veterinary Medical Association.

**Published:** 1901-10

**Authors:** W. H. Dalrymple

**Affiliations:** Baton Rouge, La.


### THE JOURNAL

OF

### COMPARATIVE MEDICINE AND VETERINARY ARCHIVES.

Vol. XXII. *OCTOBER, 1901.* No. 10.

### ANTHRAX AND PREVENTIVE INOCULATION IN LOUISIANA.[1]

By W. H. Dalrymple, M.R.C.V.S.,
BATON ROUGE, LA.

I think I can truthfully say that the control of anthrax and the possible eradication of the infection are the most profound problems that confront the agriculturist and stockowner, as well as the veterinary profession in Louisiana and the contiguous States. Such an unfortunate condition as has existed and at present exists with us is, to my mind, solely due, up to within recent years, to ignorance of the true nature of the disease and of the most effective sanitary measures for its control.

It is unnecessary to go into the ancient history of anthrax. All of you who keep up with our fatal animal plagues must be perfectly familiar with the historic division of the subject. We may say, however, that anthrax or charbon, as the French term it, is one of the oldest diseases known to medical science, and is almost universal; that it is exceedingly dangerous and fatal both to humanity and the lower animals (past records showing frightful devastation through its ravages); and that it is brought about by the introduction into the economy of the spore-bearing organism the bacillus anthracis, the specificity of which was, I believe, first recognized by Davaine in 1863.

I will not occupy your time with the bacteriology of the disease. Suffice it to say that the organism belongs to the spore-bearing variety; that it is aërobic; that the bacilli only are found in the blood, where they multiply by fission—*i. e.*, elongation and division into segments; that sporulation takes place outside of the body

[1] Read before the Thirty-eighth Annual Meeting of the American Veterinary Medical Association.

when bacilli-laden blood is exposed to the atmospheric oxygen; that the spores are more resistant to external, germicidal, and other influences, and that they will remain for a great length of time in ordinary external surroundings—and possibly vegetate there—and be capable of causing infection in man and animals when introduced into the system by different channels and through the intermediation of various agencies.

The channels by which the organism gains entrance to the circulation are generally recognized as three, viz., the alimentary tract, the skin, and the lungs. I have seen it recently stated that "authorities without exception were agreed that the almost exclusive method of infection in cattle was by taking in the germs adhering to food or in drinking-water." This may hold good in some countries, but the opinion does not altogether agree with my own experience in Louisiana.

True, I believe that first cases result from infection in this way, especially where no precautions have been taken to properly dispose of anthrax carcasses in previous years, which, unfortunately, has been the case in my State. But I think there can be no question that the different varieties of horse-flies (tabanidæ) and other blood-sucking insects are more responsible than any or all other agencies combined in spreading the disease in the lower Mississippi Valley. Of course, the first cases are responsible in providing the source from which these hordes of insects obtain the virulent material for inoculation.

Let us devote a few seconds to the consideration of a case that is an early victim of the disease and which has been left exposed on the surface of the ground, and see how we can charge responsibility of spread to such a source. At and immediately after death the blood is simply swarming with anthrax bacilli. It is a common occurrence in Louisiana to witness thousands of blood-sucking flies loading up on the virulent blood at this time, and it is almost immediately succeeding such occurrence that we begin to find numerous cases of anthrax of the external or carbuncular form, and at points widely separated, the tabanidæ, as you are no doubt aware, being exceedingly strong on the wing, and capable of flying immense distances.

On account of rapid decomposition of the body and the evolution of gases after death, we have bloody discharges issuing, probably by pressure from within from the natural openings. This virulent material exposed to the air permits of the bacilli contained within it undergoing the process of sporulation, contaminates the surroundings with which it comes in contact, besides becoming a source from which other varieties of fly scavengers, which do not puncture the skin, can obtain the virus on their mouth-parts and feet, and are then capable of transmitting the infection to susceptible animals having abrasions of the skin. Grass, herbage, and other food materials grown upon the soil contaminated by the charbonous discharges from the dead animal may and often do, as has been proved in many instances, become infected by the adherence of spores to these materials, and cause outbreaks not only in the vicinity of the victim, but wherever such foodstuffs may be transported. Virulent discharges from the carcass that have been washed by rains into running water or streams may not only contaminate the water-supply of livestock in the immediate neighborhood, but by receding, after a freshet, infect the grazing along the banks of such watercourses. Our valuable scavengers, the buzzard and the carrion crow, are no doubt responsible for spreading anthrax infection from the dead animal, for, after soiling their feet by walking over the blood and offal, as well as by other means, they are capable of producing fresh centres of the disease on the grass of fields and other places on which they alight. Hogs or swine, many of which with us are not under the immediate control of their owners, spread the infection by, first of all, contracting the disease themselves, as well as carrying infection on their feet and snouts, then dying at some distance away and creating new foci and sources from which more virulent blood may be obtained. Similar allusion may also be made to the wandering cur-dog.

These are some of the commoner agencies with us by which infection is spread from the early victims or first cases left unburned or uninterred, which, however, does not include the skinning of carcasses through ignorance of the danger to the operator, or of distributing the infection by such procedure. But, in addition to the carcasses of the domestic animals as sources of the virus, we occasionally have, in extensive epizootics, some of the wild animals, such as deer and others, in our swamps and woods, becoming affected, and thus enlarging the infected areas.

Such, I may say, has been the condition of affairs with regard to anthrax in Louisiana. For how long no one knows, but, at all events, from a time antedating the recollection of our oldest inhabitants.

Fortunately, I am pleased to be able to say, the situation is beginning to show marked evidences of improvement, as the result,

I presume, of a persistent effort to inform our people concerning the true nature of the disease, how it may reasonably be prevented, and the great importance to be attached to strict sanitary measures for its control and possible eradication, although the latter is improbable in the near future, owing to permanently infected areas and other unfavorable existing conditions.

The internal form of anthrax is, of course, produced by the ingestion of food or water infected with the specific organisms of the disease.

External or carbuncular anthrax can be brought about through any medium by which the infective germ is brought in contact with the superficial circulation or absorbent vessels. Many of these may be readily imagined, but there are one or two recorded cases in man that may be of special interest. A year or two ago a case of death from malignant pustule was reported in which an employé of the London general post-office became infected through an abrasion or sore on his hand while handling a piece of leather out of which he was making box-hinges. And the fact has been established, I believe, that even the ordinary tanning process now in vogue is not sufficient to destroy the virulence of the anthrax spore. I have observed, also, in an English medical journal or magazine that one or two boys in one of the manufacturing towns succumbed to this disease as the result of cleaning the parts of a carding-mill, they at the time having cuts or wounds on their hands. Evidently the mill must have had wool from charbonous sheep previously passed through it.

It is to the horse-fly or gad-fly, however, that I desire to make special allusion as being, in my opinion, the most potent factor in the spread of carbuncular anthrax in Louisiana. No one who has not been an eye-witness can have the most meagre conception of the appalling numbers of those tabanidæ during certain seasons in what might be termed the anthrax districts of our State. I understand there is something like five hundred species of this family of flies, and that about one hundred and fifty of them occur in North America. Porchinski, a Russian entomologist, who has made some study of the life-history and habits of this hitherto somewhat neglected order of insects, states that "water and arboreal plants are the chief conditions of the existence and multiplication of the family to which horse-flies or gad-flies belong, and where these conditions are absent no tabanidæ are observed." All such favorable conditions we possess in abundance in the sections of Louisiana which suffer most from anthrax. For instance, at the back of many of our plantations are woods and moist places, such as swampy lands, and it is to these uncultivated portions of the properties that the remains of all animals have been committed. All carcasses have been treated alike, whether charbonous or otherwise, viz., dragged or hauled out to the "bone-yard," and there left exposed on the surface of the ground. This practice has been in operation almost from time immemorial (although it is now changed for the better in most places), with the result that infection has been yearly added to the surroundings. Now, we find that first cases usually occur among animals, frequently cattle, in the neighborhood of these infected areas. With the development and multiplication of the flies, and the blood of the first victim at hand to feed upon, it may readily be inferred how the infection is scattered broadcast.

It has generally been observed that outbreaks of anthrax in epizootic form in Louisiana usually succeed protracted seasons of drought in summer and after the breaking of such drought by the first few showers of rain. On the other hand, the disease rarely occurs over an extended area, and, if at all, in only sporadic or enzoötic form, during seasons in which we have frequent and copious precipitation. This may be accounted for, first of all, by the fact that a lengthened dry spell of weather favors the development and multiplication of greater numbers of horse-flies, many of which would be destroyed in the oval or larval stages by incessant heavy rains during these more delicate stages of the insect's life.

Then, again, the moisture from the showers, following the dry weather combined with the natural heat of our summers, brings about conditions favorable to the development of latent bacterial life already in existence in infected localities.

When heavy and frequent rains continue during our summers we seem to have fewer of these flies, for the reasons, no doubt, just stated, and it is reasonable to presume that a great deal of the infection is washed from off the surface of the ground and of the vegetation, and carried away by running water, as streams, rivers, etc. This, of course, creates a menace to territory below and through which such water passes. As an instance of this we got infection on the pasture of our State Experiment Station through the discharges of a charbon victim, belonging to a neighbor immediately above us, being washed into a branch which runs through it. And the lands of our State bordering on the Mississippi River might easily be infected from the State of Mississippi to the north of us, as I understand numbers of the victims of the recent terrible epizoötic there, before the authorities took action in the matter, were thrown into the river to float down.

The third mode of infection is by way of the lungs or respiratory tract, but, although the human subject contracts anthrax as "wool-sorter's disease" by inhaling the desiccated spores from the wool of sheep that have been soiled with infected blood, I do not think that animals often receive infection in this way, and, if so, it is of somewhat rare occurrence. At all events, I should consider this mode quite infrequent as compared to the others mentioned.

(To be continued.)

